# Monitoring students’ well-being through journal analysis—study protocol of an explorative approach using natural language processing on typed and transcribed entries to monitor and generate personalized feedback

**DOI:** 10.3389/fdgth.2026.1863876

**Published:** 2026-08-28

**Authors:** Nadine N. Schmitt, Michelle D. Schlicher, Andreas Triantafyllopoulos, Lennart Seizer, Caterina Gawrilow, Carsten Eickhoff, Björn W. Schuller, Johanna Löchner

**Affiliations:** 1 Department of Child and Adolescent Psychiatry, Psychosomatics and Psychotherapy, University Hospital, Tuebingen, Germany; 2 German Center for Mental Health (DZPG), Tübingen, Germany; 3 Institute of General Practice and Family Medicine, LMU University Hospital, Munich, Germany; 4 Department of Clinical Psychology and Psychotherapy for Children and Adolescents, Friedrich-Alexander-Universität Erlangen-Nürnberg, Erlangen, Germany; 5 CHI—Chair of Health Informatics, TUM University Hospital, Munich, Germany; 6 MCML—Munich Center for Machine Learning, Munich, Germany; 7 Department of School Psychology, Eberhard Karls University Tuebingen, Tuebingen, Germany; 8 Institute for Bioinformatics and Medical Informatics, Eberhardt-Karls-Universität Tuebingen, Tuebingen, Germany; 9 MDSI—Munich Data Science Institute, Munich, Germany; 10 GLAM—Group on Language, Audio, & Music, Imperial College, London, United Kingdom

**Keywords:** feedback, journaling, monitoring, natural language processing, personalization, user engagement

## Abstract

**Background:**

More than one-third of German students report high emotional exhaustion. One effective and low-cost method for improving emotional well-being is journaling, and advances in artificial intelligence (AI), particularly in Natural Language Processing (NLP), enable automated analysis of journal content for mental health monitoring. These analyses can be returned to users as personalized feedback, thereby enhancing the positive effects of journaling through increased self-reflection and creating an incentive for continuous use, which, in turn, improves monitoring. Journaling apps offer various input modalities (e.g., typing, speaking), potentially further increasing participation. Despite the promising potential of AI-powered journaling apps, their effects on mental health have so far been scarcely investigated.

**Research question:**

This study investigates the performance of NLP models in predicting emotional well-being from journal entries. Specifically, it examines whether typed or spoken entries provide a more suitable input modality for these predictive models. Additionally, we explore whether receiving feedback is positively evaluated and which types of feedback students prefer.

**Method:**

In a two-week observational study, N=100 university students (aged 18 years and older) will be recruited and randomly assigned to one of two groups (speaking vs. typing). Following a baseline assessment, participants will submit daily journal entries and annotate them using reflective questionnaires on emotional well-being, stress, and journal topics. This information will be presented to participants as personalized feedback. Additionally, the performance of NLP models in predicting emotional well-being from journal entries will be evaluated separately for the two groups.

**Expected results:**

We expect that emotional well-being can be predicted from journal entries using NLP, with transcribed entries yielding higher accuracy than typed entries. We also expect the feedback to be well-received.

**Discussion:**

This study explores an accessible and engaging journaling application for monitoring and providing feedback on emotional well-being. It addresses several challenges in e-health research (e.g., high dropout rates, low user engagement). If this innovative approach yields strong user engagement and predictive performance, future work should evaluate automatically generated NLP-based feedback in journaling interventions to promote mental health.

**Clinical Trial registration:**

identifier DRKS00034660.

## 1 Introduction

University students in Germany experience high levels of emotional distress, with 37% reporting emotional exhaustion [1]. Emotional exhaustion is a key risk factor for mental disorders and contributes to high dropout rates in higher education [2]. Stress plays a major role in deteriorating well-being [3], increasing the risk of mental health disorders [4], and impairing academic performance [5]. Additional factors such as poor physical health, sleep problems, anxiety, and loneliness further aggravate vulnerability [1]. Despite available interventions, such as Cognitive Behavioral Therapy (CBT) [6] and mindfulness practices [7], many students lack awareness and effective coping strategies [8, 9].

Journaling represents a low-cost, accessible, and increasingly popular practice among students, with social media trends further enhancing its appeal. Empirical evidence indicates that journaling can reduce anxiety and depression [10, 11], increase resilience [12], and foster acceptance of negative emotions [13]. For students, journaling has shown benefits in reducing stress [14], improving motivation [15], and enhancing academic performance [16]. However, effects vary depending on journaling type, duration, and population [17]. Journaling can also serve as a tool for monitoring emotional well-being. Traditional self-report instruments capture only limited aspects of subjective experience, while journaling allows for a richer and more ecologically valid picture of daily emotions and stressors [18]. This makes digital journaling particularly suitable for longitudinal monitoring in naturalistic settings. Compared to social media data, which has increasingly been used for mental health prediction [19], journaling allows for greater control over timing and context, as well as structured linkage to daily self-reports, providing a ground truth that has been noted as a persistent challenge in social media-based approaches [20]. Additionally, journaling carries a dual role, serving both as a monitoring tool and as an intervention shown to contribute positively to mental health.

Journaling apps are among the most downloaded mental health apps [21, 22]. Features typically include logging of text entries, mood tracking, reflection prompts, and visualization of entries. Some apps incorporate AI features such as chatbots or automated text analysis, but these remain rare and lack scientific validation [23]. To date, few apps have been tested in controlled studies, and evidence on effectiveness and user preferences is scarce [24, 25].

NLP enables automatic assessment of emotions from text. Applications include monitoring depression severity [26], detecting suicidal tendencies [27], and tracking stress [28]. Transformer-based models such as BERT or RoBERTa are state-of-the-art for sentiment analysis and mental health prediction tasks [29–31]. However, research on NLP applied to journaling data is limited. Existing work has mostly used lexicon-based methods [32, 33] or simple polarity classification [34], which fail to capture nuanced emotional states. No studies have yet applied advanced NLP architectures to journal data. This is furthermore hindered by data scarcity. Available datasets are small, predominantly in English, and lack continuous well-being labels or multimodal entries [35, 36]. German-language data are not publicly available. However, transfer learning offers a way to address these limitations by fine-tuning pre-trained models on small, domain-specific datasets [37, 38].

Input modalities are of further concern, as digital journal entries may be spoken or typed nowadays. This may influence the linguistics, as well as the model performance, as written text is more deliberate and structured [39], while spoken language contains paralinguistic cues such as prosody and pitch that can enhance emotion recognition [40, 41]. A previous study suggests that written text and transcribed speech may yield different prediction accuracies [42], but substantial research on the effect of the input modality on model performance is missing; especially in the context of emotional expression, such as in journaling.

Feedback on the journaling content takes this digital intervention one step further, as feedback on emotional well-being can promote self-awareness, reflection, and behavior change [43, 44]. However, existing apps mostly provide simple statistics, and little is known about which feedback formats are most effective or preferred by students. Personalized feedback is also associated with improved adherence to digital health interventions [45]. Adherence, however, is further moderated by individual self-control, with higher dispositional self-control linked to stronger health behavior adherence [46]. Offering flexible input modalities (typing or speaking) may lower barriers and improve engagement [21]. Consequently, when aiming to promote consistency in journaling, the individual self-control, as well as the input modality, should be under further research.

## 2 Research questions and hypotheses

Building upon the illustrated gaps in current research, this study investigates the potential of NLP applied to digital journaling data with the aim of monitoring and supporting students’ emotional well-being, while generating individualized feedback on the journaling content and evaluating the students’ preferences.

Primarily, we hypothesize that (RQ1) emotional well-being and stress levels can be predicted from journaling entries using NLP. Prediction accuracy is expected to be higher for spoken entries (transcribed speech) compared to typed entries. Secondly, we hypothesize that (RQ2) the participants will evaluate the individualized feedback positively, indicating its acceptability and potential to foster reflection and compliance. Thirdly, we expect (RQ3) higher *state self-control* and *trait self-control* to be associated with greater adherence to the study protocol, and will be negatively correlated with dropout rates.

Furthermore, we will undergo (RQ4) an exploratory analysis of the improvement of the students’ emotional well-being over the study period, including the difference when typing vs. speaking the journal entries. Lastly, (RQ5) the predictive performance of NLP models will be compared to that of human experts (psychotherapists and clinical psychologists) in an exploratory manner to gain a practical perspective on the models’ performance. If RQ1 and RQ2 should hold true, future work should proceed to explore the usage of NLP to provide feedback automatically.

## 3 Materials and method

The study was approved in September 2024 by the Ethics Committee of the Medical Faculty of the Eberhard Karls University and the University Hospital Tuebingen (Ref. No. 464/2024BO2, Gartenstraße 47, 72074 Tuebingen, Germany) and complies with the latest version of the Declaration of Helsinki [47]. The study was registered in July 2024 (16.07.2024) in the German register for clinical trials (DRKS-ID: DRKS00034660).

### 3.1 Design

**Figure 1:**
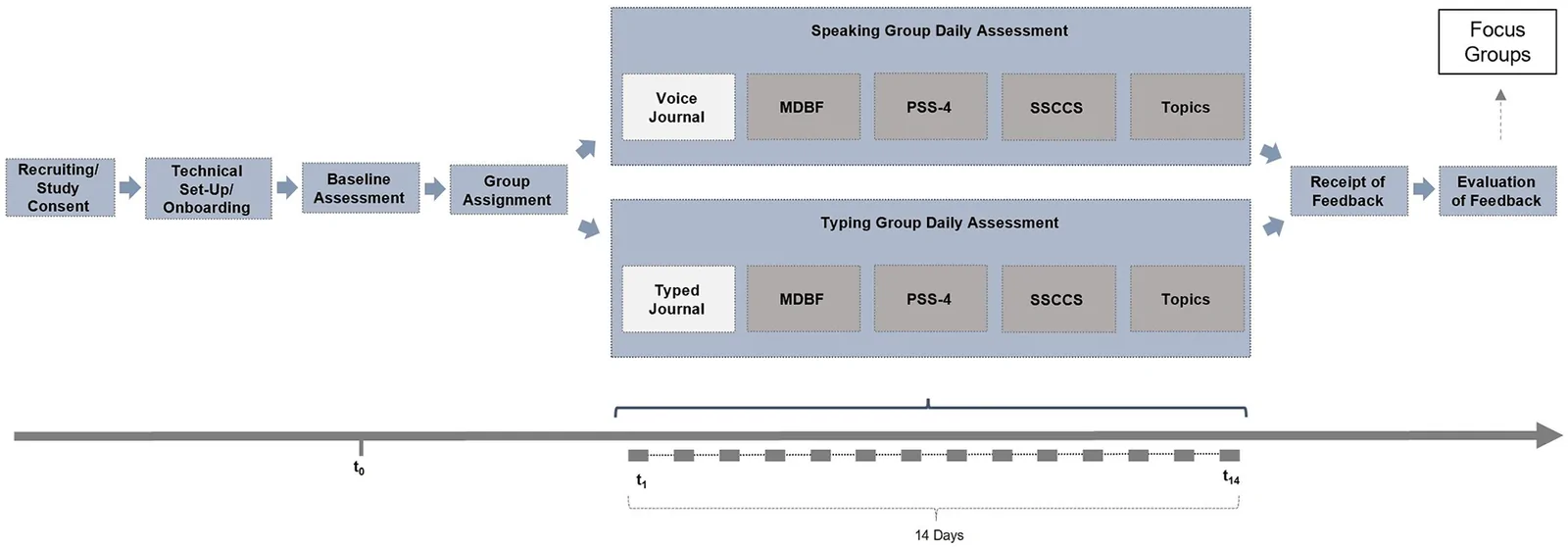
Overview of the study design.

The study is a two-week observational study with an exploratory approach that uses NLP to explore the applicability of NLP-based analyses of journal entries for monitoring students’ emotional well-being and giving individual feedback. For this purpose, we will analyze the usage of spoken vs. typed journal entries for identifying mood and stress and determine which types of feedback are favored. In more detail, the study will investigate which type of input modality performs better in predicting the students’ emotional well-being using self-labeled journaling entries as ground truth. An overview of the study design is shown in Figure 1; further procedural details are provided in Section 3.3.

At the start of the study, participants are randomly assigned to one of two groups: one group records voice messages (audio journals), while the other types their journal entries. We aim to recruit N=100 students aged 18 years or older. Once the technical setup and baseline assessment are completed, participants will provide daily journal entries and complete reflective questionnaires in their everyday lives. These questionnaires will capture information on emotional well-being, stress levels during the day, as well as state self-control, and the topics addressed in the journaling entries. All questionnaire evaluations are considered to be ground truth values, which means that they are the actual reference values for training and evaluating the models. To enhance motivation and facilitate reflection, participants will receive personalized and comprehensible feedback based on their data at the end of the 14-day period. This feedback is derived exclusively from the collected self-report data and does not incorporate any NLP-based analysis to avoid the risk of misinformation. As a final step, participants will be asked to complete a questionnaire that evaluates the feedback document. Additionally, focus group sessions will be held with voluntary participants to obtain qualitative insights into their experiences and study-related feedback (see Section 3.6). Lastly, to contextualize the performance of the NLP models, their predictive accuracy will be compared to that of human experts (psychotherapists and clinical psychologists), thereby providing a practical benchmark for model evaluation (see Section 3.7). No pilot phase will be conducted.

### 3.2 Participants and recruitment

Eligible participants will be university students who (a) are at least 18 years of age, (b) possess an internet-enabled smartphone (iOS or Android), and (c) demonstrate adequate proficiency in German, defined as fluent comprehension and production in both spoken and written form. Exclusion criteria are intentionally kept to a minimum to promote broad accessibility. Participants will be excluded only if they are experiencing an acute mental health crisis that necessitates immediate clinical intervention (e.g., acute suicidality).

To minimize participant attrition, several incentive mechanisms will be implemented: (1) all participants will receive individualized feedback upon completion of the 14-day study period; (2) participants who successfully complete the study will be compensated with a €10 voucher redeemable at various online retailers; (3) three additional €50 vouchers will be allocated by lottery among participants within the upper 50% of compliance; and (4) psychology and cognitive science students at the University of Tuebingen will be eligible to obtain university-internal credits.

A non-probability convenience sampling strategy is applied. Recruitment will be carried out through several complementary channels. Primary outreach will occur via university-wide email distribution lists at the University of Tuebingen, where psychology and cognitive science students will be explicitly informed that participation qualifies for university-internal credits. Students from all other disciplines will likewise be eligible and encouraged to participate. Additional on-campus recruitment will be conducted through the display of posters in university buildings. Digital recruitment will include dissemination of study information via the official social media account of the University Hospital Tuebingen and the extension of outreach to students at other universities through targeted posts in messenger-based student groups.

**Figure 2:**
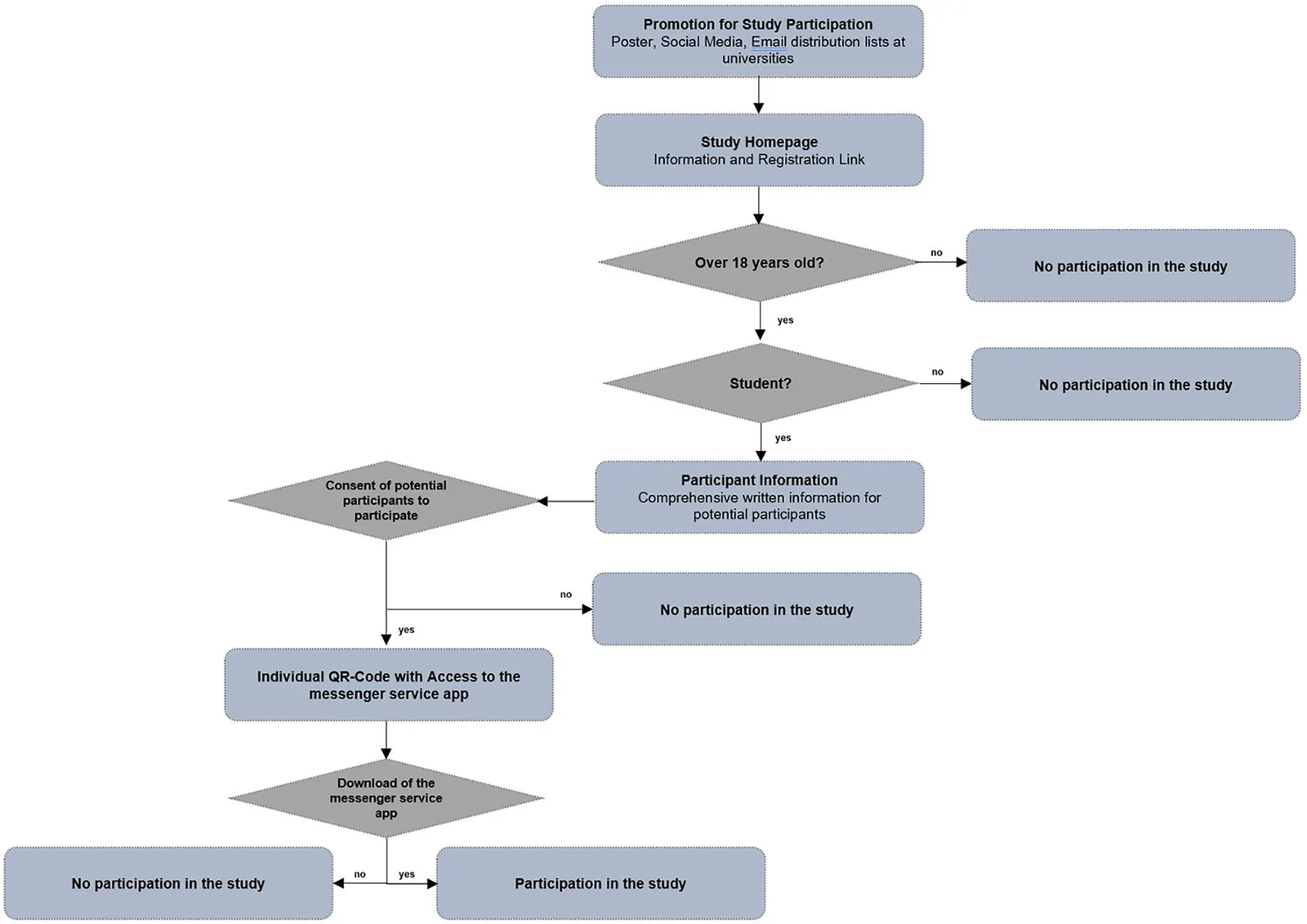
Registration process.

During recruitment, participants will be directed via QR code to the study homepage,^1^ where they can access detailed study information and complete the online registration. Figure 2 provides an overview of the registration process. Participants accessing the registration link will first be asked to confirm that they meet the eligibility criteria; individuals who do not meet these criteria will not be able to proceed. Eligible participants will then provide informed consent by affirming the relevant checkboxes. Using the contact information provided, the study team will send an email containing a personalized QR code directing participants to download a GDPR-compliant messenger service, which will be used to maintain contact with participants throughout the study and to manage data collection. The messenger service facilitates secure, direct communication between participants and the study team, allowing participants to submit individual inquiries via chat, which will be answered on a daily basis. The email will also include an instructional video guiding participants through the installation process and explaining the app’s functionalities. The personalized QR code grants each participant access to a unique user account linked to an individual identifier within the app. Once the application has been installed and activated, the participant will be formally enrolled in the study.

### 3.3 Procedure

Upon completion of enrollment, participants will be welcomed by the study team within the app and prompted to complete the baseline assessment questionnaires. For this purpose, they will receive an individualized link via the messenger service to an online survey hosted on the SoSci Survey platform^2^, which can be accessed through their smartphone browser. The link will contain each participant’s unique code to ensure accurate data assignment. The survey will cover demographics, education, general well-being, mental health, stress, and dispositional self-control, and is expected to take no longer than 10 to 15 min to complete. Subsequently, participants will be randomized into two groups using block randomization with randomly varying block sizes of 4, 6, and 8 to ensure approximately equal distribution. To generate the list of group assignments, the tool Sealed Envelope [48] is used. Participants are then assigned based on their registration sequence. The type group will be instructed to consistently type their journal entries, whereas the speak group will be instructed to record their journal entries as voice messages via the messenger service. In order to establish a “neutral voice” baseline for each participant in the speaking group, they will be asked to read standardized text snippets from the literature on voice assessment.^3^ These snippets are designed to avoid eliciting emotion or cognitive load.

During the 14-day study phase, participants will be asked to record a daily journal entry as a (voice) message in the messenger service app, sharing their “high- and low-lights” of the day by either typing or speaking according to their group assignment. They will receive a notification via the messenger service around 6 p.m., but the assessment may be completed at any time during the remainder of their day. Immediately after creating the journal entry, participants will be asked to complete a short reflective survey on the SoSci Survey platform, accessed via an individualized link provided in the reminder message. Since the links will be personalized, all reminders will be sent individually and manually by the study team. The sequence—first journaling, then completing the questionnaire—is chosen to ensure that participants can report naturally on topics they consider relevant, without potential bias introduced by answering questionnaires beforehand. All questionnaire scores will serve as ground truth reference values for training and evaluating the NLP models. The total time required for journaling and survey completion is expected to be approximately 5–10 minutes per day. Each daily data point will include a journal entry, three emotional well-being metrics, a daily stress level, a measure of situational self-control, and a list of topics mentioned in the journal entry. To enhance compliance, the study team will monitor on a daily basis whether participants have completed the required assessment. If an assessment is missed or remains incomplete from the previous day, a reminder will be sent in the afternoon notifying the participant of the missed entry. The reminder will also include a brief question regarding the reason for the omission, along with an encouragement to complete the assessments on subsequent days. After the two-week study period, participants will receive individualized feedback based on their journal entries and questionnaire responses (see Section 3.5 for more detail). Providing it once at the end of the study was intended as an incentive to complete the two weeks. As a final step, participants will be asked to complete a feedback questionnaire to provide direct evaluations of their study experience. In addition, focus group sessions with voluntary participants will be conducted to further explore participant perspectives (see Section 3.6 for further details).

### 3.4 Assessment instruments

In the following, all assessment instruments are described in detail. There are three different sets of assessment instruments in this study: (1) the baseline survey completed by each participant prior to the start of the study; (2) the journaling entries completed at each measurement time point during the 14-day assessment period; and (3) the daily surveys completed at each measurement time point during the 14-day assessment period, which serve as ground truth for predicting the content of the journaling entries (target vaiables).

#### 3.4.1 Baseline survey

Prior to the start of the study, participants will complete several baseline questionnaires: a demographic questionnaire capturing medication use and health history (see Additional file 1 of the Supplementary Material to this article), an educational level questionnaire based on the International Standard Classification of Education—Fields of Education and Training 2013 [49], and the WHO-5 Well-Being Index (WHO-5) [50] assessing general well-being. In addition, mental health and stress will be evaluated using the 21-item Depression Anxiety Stress Scales (DASS-21) [51], while dispositional self-control will be measured with the Brief Self-Control Scale (SCS-K-D) [52]. All instruments were administered in their validated German versions.

#### 3.4.2 Daily journaling

During the 14-day study period, participants will be prompted each evening via the messenger service to record a voice message reflecting on the day’s highlights and lowlights. The standardized instruction will be: “What were your high- and lowlights of the day? How did they make you feel and why?” This prompt is intended to facilitate reflection on both positive and negative experiences. In contrast to stream-of-consciousness instructions (e.g., “Write about your day”), which may yield brief responses, this question is expected to elicit more elaborated entries. By addressing overall emotional well-being, it allows for a broad spectrum of emotions to be captured, rather than restricting the focus to a single emotional dimension as in approaches such as gratitude journaling or expressive writing. No constraints will be imposed on entry length, thereby enabling participants to provide responses of natural scope without artificial extension.

#### 3.4.3 Daily target variables

The daily surveys will include the Multidimensional Mood State Questionnaire (MDBF), a parsimonious six-item mood scale designed for momentary assessment in daily life to evaluate participants’ well-being [53]. This instrument is derived from the German-language Multidimensional Mood Questionnaire (MDMQ) [54] and captures three dimensions: valence (good–bad mood), arousal (wakefulness–fatigue), and calmness (calmness–restlessness). Scores range from 1 (bad mood, fatigue, restlessness) to 7 (good mood, wakefulness, calmness). In addition, the ecological momentary assessment version of the Perceived Stress Scale-4 (PSS-4) [55] will be administered to assess daily stress levels. Both the MDBF and the PSS-4 will be adapted to refer to the entire past day rather than the immediate moment of completion. This adaptation will ensure consistency with the journaling entries, which also cover the whole day, thereby enabling the questionnaire scores to be meaningfully predicted from the entries by the NLP models. Furthermore, participants will answer two questions from the State Self-Control Capacity Scale (SSCCS) [56] to assess their situational self-control when journaling.^4^ Finally, participants will also select from a predefined list the topics mentioned in their journal entry. The possible topics are adopted from a North American study on diary content [57] and translated into German: movement, family, food, friends, religion/spirituality, health, love, recreation, school, sleep, and work. The term “religion/spirituality” will be used instead of “god,” as religion is practiced less centrally in European countries compared to North America [58].

### 3.5 Individual feedback

After the two-week journaling assessment period, participants will receive written feedback of their provided data in the form of a PDF document (see Additional file 2 of the Supplementary Material to this article) delivered via the messenger service, based on their completed psychopathological questionnaires and journaling entries. The feedback will be generated with Python and include the following elements:

- Overview of the demographics describing the study population.
- Explanation, evaluation, and interpretation of the baseline questionnaires. Since the WHO-5 and the SCS-K-D do not provide a direct interpretation of their scores, averages from the German population were provided for comparison.
- A heat map and a line chart visualizing the MDBF values over time; the former based on the original scale of the questionnaire and the latter centered on the individual mean. Person-mean centering will be applied to make the questionnaire scores more interpretable for participants, with 0 reflecting each participant’s average for a given dimension. Negative scores will indicate worse-than-average scores, while positive scores will indicate better-than-average scores.
- A line graph visualizing the PSS-4 score over time, centered on the individual mean.
- A pie chart visualizing the distribution of the mentioned topics.
- Four times a bar chart showing the valence values of the MDBF over time, while centered on the individual mean. Individual charts will be colored according to the occurrence of the four most-mentioned topics. If the topic is mentioned on a day, the bar will be colored orange; otherwise, it will be colored gray. This should allow identifying relations between valence and topics.
- Three word clouds: one over all journal entries, one with words from the days with positive MDBF valence scores centered on the individual’s average, and one from the negative days. This should allow insights into predominant topics for better and worse days. In a pre-processing step, stopwords will be removed using Natural Language Toolkit (NLTK) [59].
- All (transcribed) journal entries with date and time.
- The feedback will conclude with a list of contact addresses for support services and assistance, and participants will be reminded that they may contact the study team at any time with questions regarding the feedback. In addition, participants whose DASS-21 scores exceed the respective cutoff values and who meet the age eligibility criteria will receive information about an additional study that offers access to psychological counseling.

It should be noted that the feedback is based solely on each participant’s self-report data and does not incorporate any insights derived from the NLP analyses. This approach ensures that participants receive feedback that is independent of AI and remains accurate and meaningful even if model performance is suboptimal. The goal is to evaluate the capability of NLP models to infer indicators of users’ mental health (mood and stress), such that future studies may incorporate automatically generated feedback that includes these assessments. Should participants have questions about the feedback or feel uncertain or distressed by any of the information provided, they will be able to contact the study team for clarification and support.

### 3.6 Participants involvement

As part of a participatory research approach, students will have the opportunity to provide direct feedback to the study team in order to evaluate the different types of feedback, indicate their preferred input modality, and critique the study. For this purpose, participants will be asked at the end of the study to complete a feedback questionnaire, which is provided in the Additional file 1 of the Supplementary Material to this article. Responses will be recorded using a 7-point Likert scale ranging from 1 (not at all) to 7 (very much)^5^. The questionnaire will consist of 23 quantitative items and 19 open-ended questions, all of which will be mandatory.

To gain a deeper understanding of participants’ opinions and to obtain richer insights into their attitudes, experiences, and perspectives regarding their motivation, needs, and potential barriers related to the study, two guided qualitative focus group interviews with three to four participants will be conducted. The participation will be voluntary. For this purpose, a focus group guideline (see the Additional file 3 of the Supplementary Material to this article) will be collaboratively developed within the research team. The guideline will address several relevant topics, including motivation for study participation, reflections on the scope and feasibility of the assessments, completion of questionnaires, individual feedback after measurement points, the anticipated impact of the study on daily life, and suggestions for possible modifications to the study design.

### 3.7 Expert rating

To put the NLP model performance into relation to human performance, we will implement an online survey using the SoSci Survey platform for exploratory comparison. Licensed psychotherapists and clinical psychologists will be recruited via email invitations. Each participant will evaluate the well-being and stress levels of 24 anonymized journal entry samples by estimating the three MDBF subscales and the PSS-4 score. The number of items are chosen to keep the completion time around 45 min (approx. 1.5 min per entry). The presentation order will be randomized between participants. Ratings will have to be made on continuous sliders (one-decimal precision), ranging from the minimum to maximum values of the respective questionnaires. This format ensures direct comparability with the model predictions, which also yield decimal outputs. As an incentive, participants will receive feedback at the end of the study: the journal entries will be presented again, along with their corresponding ground truth values, allowing the experts to compare their own assessments to the actual scores.

### 3.8 Participants’ support and guidance

Throughout the study period, participants will receive technical and psychological support if needed and the option to ask questions via the messenger service. Any adverse events and negative effects will be systematically recorded. Since the target group partly consists of at-risk participants with elevated psychological burden, close monitoring and accessibility in the event of psychological crises or other incidents are crucial, making intensive support essential. In the event of unexpected incidents that pose an acute risk to participants due to study conditions, the entire study will be immediately terminated. Participants can contact the study team at any time in such cases and opt out of the study at any time.

## 4 Statistics

### 4.1 Sample size calculation

In this NLP study, classical power analysis is not directly applicable, as the model performance depends on several factors such as training data points, model architecture, and transfer learning effects. Therefore, based on preceding transfer learning literature, 500 data points (D) per group (type and speak) are targeted [60, 61]. Each participant is expected to contribute one data point daily over the study duration of 14 days (d). A dropout rate of 28% (r) is assumed based on comparable studies [62–64]. The number of participants is calculated to be 50 participants per group (100 total):

$$N=\frac{D}{d\times (1-r)}=\frac{500}{14\times (1-0.28)}\approx 49.6\approx 50$$

### 4.2 Statistical analysis

#### 4.2.1 Baseline assessment

Baseline questionnaire data will be summarized descriptively, and Spearman’s rank correlations will examine relationships between DASS-21 subscales, WHO-5, and SCS-K-D scores. Differences by sex and randomized group (type vs. speak) will be explored using two-way ANOVA. *P*-values will be reported for reference, but the study is not powered to detect baseline differences.

Daily measures of mood (MDBF subscales), stress (PSS-4), and state self-control (SSCCS) will be analyzed using linear mixed-effects models with random intercepts for participants to account for repeated measurements. Baseline well-being measures (SCS-K-D, DASS-21, WHO-5) and group assignment will be included as fixed effects to explore whether baseline characteristics predict daily mood and self-control. Daily observations will be included only for days with completed journals; the models naturally handle these unbalanced repeated measures. Additionally, person-level averages of daily mood and state self-control may be correlated with baseline measures using Spearman’s rank correlation as a descriptive check.

#### 4.2.2 RQ1: NLP analysis

The spoken journal entries will be transcribed using Whisper [65]. All journal entries will be preprocessed according to standard NLP procedure (tokenization, lowercasing, lemmatization, and stop-word removal) to generate sparse representations [e.g., term frequency–inverse document frequency; TF–IDF). Additionally, dense representations (e.g., contextual embeddings from German pre-trained transformer-based models like GBERT [66] or German-BERT [67]] will be generated. Here, preprocessing will be limited to the model-specific tokenization procedure.

The comparison of architecture classes is itself part of the study, as prior literature has not yet established a best-performing architecture for journaling data. Therefore, different supervised machine learning models will be employed for this regression task, ranging from classical approaches (e.g., random forest regression or support vector regression) to transformer-based deep learning models (e.g., BERT-based regression head). Separate predictive models will be trained on the above-mentioned representations for each outcome variable (MDBF subscales, PSS-4). We will additionally evaluate end-to-end fine-tuning of a smaller transformer model (e.g., a distilled German BERT variant), which will operate directly on tokenized journal entries rather than precomputed representations. Here, we acknowledge the higher risk of overfitting compared to our main representation-based approach.

As a baseline, a mean predictor will be implemented. Model performance will be quantified using root mean squared error (RMSE) and mean absolute error (MAE) to capture prediction error magnitude, and Pearson correlation to assess the strength of association between predicted and observed scores. All models will be evaluated using k-fold cross-validation. To account for repeated measures within participants, cross-validation will be conducted at the participant level, such that all entries from a given participant are assigned exclusively to either the training set or the test set. To examine group differences, models will be trained and evaluated separately for the written and transcribed groups. Predictive performance metrics will then be compared across groups. Differences in performance will be formally tested using bootstrapped confidence intervals to provide robust inference without assuming normality of error distributions.

#### 4.2.3 RQ2: feedback evaluation

Quantitative data from the feedback questionnaire will be analyzed descriptively (means, standard deviations, medians, and ranges). Open-ended responses will be explored using text-mining techniques (e.g., n-gram analysis) to identify commonly used words and phrases. Additionally, the open-ended responses and focus groups will be analyzed using Mayring’s Qualitative Content Analysis [68] with deductive and inductive category development. Categories will be predefined using the interview guideline and enriched with emerging topings from the discussions.

#### 4.2.4 RQ3: compliance and self-control analysis

Compliance will be defined as daily completion of both the journal and questionnaire (0 = not completed, 1 = completed) across the 14-day study period. Daily compliance will be analyzed using a mixed-effects logistic regression with fixed effects for group, day, trait self-control (SCS-K-D), mean state self-control (SSCCS), their relevant interactions, and a random intercept for participants to account for repeated measures. Spearman’s rank correlation will be used descriptively to assess the association between trait self-control and total journals completed.

To predict on a global level whether participants will drop out (0) or complete the study (1), a logistic regression will be performed with trait self-control (SCS-K-D) and mean state self-control (SSCCS) as predictors of study completion. The study will be considered completed if participants submit the feedback questionnaire at the end of the study.

#### 4.2.5 RQ4: exploratory analysis of well-being

To explore whether emotional well-being and stress change over the 14-day study period and whether these changes differ between the type and speak groups, a mixed-effects model will be used. Fixed effects will include group, day, and their interaction (Day × Group). Random intercepts and slopes for participants will account for within-subject variability and individual trajectories.

#### 4.2.6 RQ5: expert rating

The best-performing NLP model will be retrained with optimized hyperparameters on the full dataset, excluding the expert-rated entries. Model and expert performance will be compared on item-level absolute errors using RMSE, MAE, and Spearman’s ρ, with parametric or nonparametric pairwise tests applied as appropriate. Effect sizes and 95% confidence intervals will be reported, and bootstrap confidence intervals will supplement differences in MAE and RMSE.

### 4.3 Data management

#### 4.3.1 Handling of missing data and dropout

Incomplete daily records will not be used for analysis. Specifically, if a participant provides only a journal entry or only a daily questionnaire for a given day, that day’s data point will be excluded from the dataset. Missing data will not be imputed, as journal entries and subjective reports cannot be reliably reconstructed. Instead, the extent and patterns of missingness will be explored descriptively to assess whether data are missing at random or show systematic trends (e.g., participant characteristics, or study phase). Participant dropout will be recorded, including the timing and, where possible, reasons for discontinuation. Dropout has been accounted for in the sample size calculation.

#### 4.3.2 Data security, storage and dissemination of findings

Procedure for data management was approved by the data protection officer and the IT security officer of the University Hospital Tuebingen, Germany. The collection and storage of all data is pseudonymized and stored for 10 years. All assessment instruments are labeled with an anonymous personal ID of the participant. Data can only be assigned to individuals based on a coding list, which is continually updated by hand, kept locked, and only accessible to study management. The coding list is destroyed at the end of the study. The daily measures, including the voice recordings, are collected with the General Data Protection Regulation (GDPR/DSGVO) compliant messenger Famedly,^6^ which is ISO 27001 certified and has been specifically developed for clinical contexts. Data transfers are end-to-end encrypted. All data processing complies with GDPR/DSGVO and applicable German data protection law. No external APIs are used for data processing. The transcription software Whisper runs locally.

At the end of the study, all participant data is deleted from the servers used by SoSci Survey and Famedly. From this point on, the pseudonymized data of the participants are stored exclusively on the university hospital’s drive and the university hospital’s cloud, to which only authorized staff have access. Participants are informed about their rights and may withdraw at any time; already-collected pseudonymized data will be retained unless deletion is explicitly requested, in which case it will be removed using the coding list.

Participants will receive individual feedback on their data. The results of the study will be reported in international scientific journals and at scientific conferences in the areas of mental health, child, and adolescent psychiatry and psychotherapy, affective computing, and related areas.

## 5 Discussion

This study is the first to examine the effectiveness of NLP-based analyses of journal entries for monitoring and reflecting students’ emotional well-being. The study will investigate (RQ1) whether spoken vs. typed journaling entries influence the monitoring of emotional well-being and (RQ2) which types of feedback are preferred by students. To this end, it will be assessed which input modality is better suited to predict students’ emotional well-being and stress, with self-annotated journal entries serving as ground truth. We hypothesize that students’ emotional well-being can be predicted from journaling entries using NLP, with higher predictive accuracy for spoken compared to typed entries. We further expect that stress levels can be predicted from journaling entries, and that participants will evaluate the individualized feedback positively.

Owing to the messenger-based guidance provided throughout the study period, the individualized feedback on participants’ data, and the monetary incentives offered, high compliance is anticipated, thereby facilitating the collection of the data required for training the NLP models. In cases of temporary demotivation, participants may additionally be re-engaged through personalized messages, helping to maintain sustained participation over the course of the study. Furthermore, the provision of a low-threshold, fully online study—independent of location and infrastructure and accessible to everyone—addresses the challenge of potential bias from non-representative samples limited to non-rural areas. Ethical approval and the implementation of a robust data protection concept constitute essential prerequisites for ensuring participant trust and compliance.

Nevertheless, several important considerations must be taken into account. Primarily, journaling itself may exert intervention effects beyond its function as a data collection method. Previous research suggests that reflective writing can improve emotional awareness, support emotional processing, and promote well-being. Thus, repeated journaling over the study period may itself contribute to changes in emotional well-being and stress levels. Additionally, the personalized feedback is expected to play a central role in supporting participant motivation. It may also exert an intervention effect, as engaging with one’s emotional experiences and receiving feedback on them may foster reflection and promote a deeper understanding of daily emotional states and overall well-being. By making patterns in mood and stress explicit and visible over time, feedback may support participants in recognizing triggers and recurring patterns, knowledge that could be applied beyond the study period. Although we anticipate that the data collection procedures and personalized feedback will have generally positive effects on participants’ mental well-being, it is also essential to consider potential unintended effects of continuous self-monitoring. In certain psychiatric populations—such as individuals with eating disorders, addictions, or trauma-related conditions—frequent engagement with symptom-focused self-reports may influence the occurrence or salience of symptoms. The potential impact of such biases will therefore be further examined in the planned focus group sessions. It should also be noted that, although personalized feedback will be provided at the end of the study, no brief algorithmically triggered in-app interventions based on participants’ digital traces will be delivered. Such interventions have demonstrated beneficial effects in previous research [69] and should therefore be considered in future studies.

To maximize adherence, the participants should have the option to receive their notifications at individually chosen times. If this is not possible, a reminder closer to the common bedtime should be chosen. Furthermore, in order to investigate differences in speaking and typing, participants are randomly assigned to either the typing or speaking group. Allowing participants to freely choose their group is recommended to reduce early dropout before the study begins and to improve the journaling experience, as individuals are likely to differ in their preferred mode of expressing thoughts and emotions. This recommendation is consistent with previous studies advocating for personalized settings in mobile health applications [21]. Moreover, in cases of excessive smartphone use, participants may express a desire for off-screen time and a preference for pen-and-paper journaling over digital journaling. A potential future application could therefore involve offering pen-and-paper journaling in combination with a text recognition tool to capture journal content from photographs. Beyond this, alternative input modalities such as video recordings, drawings, or other creative formats could be considered to broaden accessibility and better accommodate individual preferences.

Finally, in order to increase the depth of information provided by feedback, more advanced feedback options should be considered in future research. Existing AI tools offer promising approaches by visualizing topics in text as connected networks and identifying gaps between them. By analyzing which topics are linked and which are not, actionable recommendations can be derived for users to bridge these gaps. For example, if “sports” and “nature” appear as unconnected topics, an outdoor sport could be suggested. Such approaches could be used to generate positive behavioral recommendations. Furthermore, large language models (LLMs) show great potential in the context of journaling [70–72]. Research is starting to explore their use in generating personalized journaling prompts based on users’ prior entries, fostering deeper self-reflection. LLMs may also be used to summarize entries and extract higher-level insights, such as recurring themes or shifts in emotional tone. Such feedback can then be delivered in a more conversational format, allowing users to engage interactively with their own reflections rather than receiving static summaries. However, the use of LLMs in mental health contexts has also been widely criticized, particularly regarding reliability and the potential for harm, given the sensitive nature of the content involved [73, 74]. Nevertheless, advanced analyses of journal entries hold great potential for providing deeper insights into users’ emotional well-being that go beyond descriptive statistics. Despite these limitations, with this study, we make a substantial contribution to closing the research gap in NLP-based journaling to enhance the emotional well-being of students.

## Footnotes

^1^https://www.medizin.uni-tuebingen.de/de/journaling-studierende (Accessed on 12 September 2025.)

^2^https://www.soscisurvey.de (Accessed on 12 September 2025.)

^3^https://www.bas.uni-muenchen.de/forschung/Bas/BasPD1Contents (Accessed on 12 September 2025.)

^4^(1) Wenn ich jetzt eine schwierige Aufgabe gestellt bekäme, würde ich schnell aufgeben. (2) Wenn ich im Augenblick durch etwas in Versuchung geführt würde, würde es mir sehr schwer fallen, zu widerstehe.

^5^German scale: “Gar nicht” (1), “kaum” (2), “wenig” (3), “mittelmäßig” (4), “etwas” (5), “viel” (6), “sehr” (7)

^6^https://www.famedly.com/ (Accessed on 03 July 2026)
