# Supplementary material for: Monitoring students’ well-being through journal analysis—study protocol of an explorative approach using natural language processing on typed and transcribed entries to monitor and generate personalized feedback
Source: Front Digit Health. 2026 Aug 28;8:1863876. doi: 10.3389/fdgth.2026.1863876 (PMC13563375; doi:10.3389/fdgth.2026.1863876)
Supplement: Supplementary file 1 [file Datasheet1.pdf]

Custom Questionnaires

1 Socio-Demographic Questionnaire

Frage	Antwortformat
Geschlecht	Männlich | Weiblich | Divers
Alter	*Freitext*
Nationalität (laut Pass)	Deutsch | Andere:
Derzeitiger Familienstand	Ledig | in Paarbeziehung lebend | verheiratet | geschieden | verwitwet
Haben Sie bereits an einer Studie teilgenommen	Ja | Nein
Körpergröße	*Freitext* cm
Aktuelles Körpergewicht	*Freitext* kg
Wurde bei Ihnen jemals eine psychische Erkrankung diagnostiziert?	Depression | Angststörung | Essstörung | AD(H)S | Autismus | Psychose | Suchterkrankung | Sonstige:
In welchem Zeitraum sind die diagnostizierten psychischen Erkrankungen aufgetreten? (Mehrfachantworten möglich)	Diagnose:, Zeitraum:
Waren Sie schon mal in einer psychotherapeutischen / psychiatrischen Behandlung?	Ja | Nein
Falls ja in welchem Setting fand diese statt?	Ambulant | Tagsklinisch | Vollstationär
Nutzen Sie ein hormonelles Verhütungsmittel?	Nein | Ja | Wenn ja, welches:
Haben Sie jemals regelmäßig Beruhigungs- bzw. Schlafmittel eingenommen (Benzodiazepine, z.B. Valium)?	Nein | Ja | Wenn ja, welche: | Wenn ja, in welchem Zeitraum:
Nehmen Sie derzeit Antidepressiva (z.B. Remergil© (Mirtazapin), Tofranil© (Imipramin), Fluox© (Fluoxetin), Zoloft© (Sertralin))	Nein | Ja | Wenn ja, welche:
Nehmen Sie derzeit Glukokortikoide (Prednisolon, Dexamethason, Cortison)?	Nein | Ja | Wenn ja, welche:
Nehmen Sie derzeit andere Medikamente ein?	Nein | Ja | Wenn ja, welches:
Wie häufig trinken Sie Alkohol?	Nie | Seltener als 1x monatlich | 1-4x monatlich | 2-3x in der Woche | 4x in der Woche oder häufiger
Wie häufig rauchen Sie Zigaretten?	Nie | Seltener als 1x pro Woche | 1-4x pro Woche | 1-2x täglich | 3x täglich oder häufiger

Frage	Antwortformat
Haben Sie bereits Erfahrung im Journaling?	Nein | Ja | Wenn ja, wie lange:

2 Questionnaire to Evaluate the Feedback Document

Frage	Antwortformat
Wie hat Ihnen das Journaling gefallen?	Gar nicht (1) - Sehr gut (7)
Haben Sie das Gefühl, dass es Ihnen durch das Journaling besser ergangen ist?	Gar nicht (1) - Sehr (5)
Wie hat Ihnen die Feedback Variante 1 - 7 gefallen?	Gar nicht (1) - Sehr gut (7)
Was hat Ihnen an dieser Feedback gefallen?	*Freitext*
Was hat Ihnen an dieser Feedback nicht gefallen?	*Freitext*
Fanden Sie das Feedback zur emotionalen Befindlichkeit oder zu den Topics hilfreicher?	Befindlichkeit | Topics | Beides gleich
Wenn Sie regelmäßig eine Journaling App nutzen würden, welches Feedback würden Sie am liebsten erhalten?	1 | 2 | 3 | 4 | 5 (Mehrfachnennung möglich)
Wenn Sie regelmäßig eine Journaling App nutzen würden, wie häufig würden Sie gerne Feedback erhalten?	Täglich | alle 2-3 Tage | Wöchentlich | Zwei-Wöchentlich
Würden Sie eher beim Journaling bleiben, wenn Sie solches Feedback erhalten würden?	Nein | Weiß ich nicht | Ja
Haben Sie andere Vorschläge oder Wünsche, was für Feedback Sie gerne zu Ihren Journaling-Einträgen erhalten würden?	*Freitext*
Wenn Sie einen Tag keinen Eintrag erstellt haben, woran lag dies?	*Freitext*
In welcher Form würden Sie am liebsten Journaling betreiben?	Stift und Papier | Tippen in einer App | Sprachaufnahmen | Singen | Zeichnen | Videoaufnahme (Mehrfachnennung möglich)
Wenn Sie regelmäßig Journaling betreiben würden, wie viel Zeit würden Sie dafür täglich aufwenden wollen?	weniger als 5 Minuten | weniger als 10 Minuten | weniger als 20 Minuten | mehr als 20 Minuten
Wenn Sie regelmäßig Journaling betreiben würden, an wie vielen Tagen der Woche würden Sie dies gern tun?	Täglich | alle 2-3 Tage | Wöchentlich

**Frage**	**Antwortformat**
Hätten Ihnen individuelle Fragen als Anleitung zum Journaling besser gefallen, als die Standardfrage zu High- und Lowlights?	Nein | Weiß ich nicht | Ja
Wären Sie dazu bereit, über einen längeren Zeitraum Journaling zu betreiben?	Nein | Weiß ich nicht | Ja
Gab es Feedback, das sich negativ auf Sie ausgewirkt hat?	Nein | Ja, welches: *Freitext*
Falls Sie vor dieser Studie bereits Journaling per Stift und Papier betrieben haben, wie unterscheidet sich dieses zum Journaling mit App und Feedback?	*Freitext*
Wenn es Ihnen schlecht gehen würde oder Sie eine stressige Phase hätten, welche Eingabemöglichkeit würden Sie bevorzugen?	Stift und Papier | Tippen in einer App | Sprachaufnahmen in einer App (Mehrfachnennung möglich)
Was sind für Sie die Vorteile des Journalings per Stift und Papier bzw. per Sprachaufnahme?	*Freitext*
Haben Sie weitere Anregung oder Kritik zum Journaling per App	*Freitext*
Haben Sie weitere Anregung oder Kritik zur Studie allgemein?	*Freitext*
